# The Role of Pyroptosis and Autophagy in Ischemia Reperfusion Injury

**DOI:** 10.3390/biom12071010

**Published:** 2022-07-21

**Authors:** Huijie Zhao, Yihan Yang, Xinya Si, Huiyang Liu, Honggang Wang

**Affiliations:** 1 Institute of Chronic Disease Risks Assessment, Henan University, Jinming Avenue, Kaifeng 475004, China; zhj5696@163.com; 2 School of Basic Medical Sciences, Henan University, Kaifeng 475004, China; h1323240458@163.com (Y.Y.); m15736875597@163.com (H.L.); 3 School of Stomatology, Henan University, Kaifeng 475004, China; 1923010012@henu.edu.cn

**Keywords:** pyroptosis, autophagy, ischemia/reperfusion injury, apoptosis, NLRP3

## Abstract

Pyroptosis is a process of programmed cell death mediated by gasdermin (GSDM) found in recent years. In the process of pyroptosis, caspase-1 or caspase-11/4/5 is activated, which cleaves gasdermin D and separates its N-terminal pore-forming domain (PFD). The oligomers of PFD bind to the cell membrane and form macropores on the membrane, resulting in cell swelling and membrane rupture. Increasing evidence indicates that pyroptosis is involved in many diseases, including ischemia reperfusion injury. Autophagy is a highly conserved catabolic process in eukaryotic cells. It plays an important role in the survival and maintenance of cells by degrading organelles, proteins, and macromolecules in the cytoplasm and recycling degradation products. Increasing evidence shows that dysfunctional autophagy participates in many diseases. Recently, autophagy and pyroptosis have been reported to play a vital role in the process of ischemia/reperfusion injury, but the related mechanisms are not completely clear. Therefore, this article reviews the role of autophagy and pyroptosis in ischemia–reperfusion injury and analyzes the related mechanisms to provide a basis for future research.

## 1. Introduction

Pyroptosis, a lytic and inflammatory programmed cell death pathway distinct from apoptosis, was first discovered in infected macrophages in 1992 and named by Cookson et al. in 2001 [1,2]. Pyroptosis is primarily induced by inflammasomes and performed by caspases, including caspase-1/-3/-4/-5/-8/-11 and the gasdermin (GSDM) protein family [3]. The formation of pores in cell membrane, cell lysis, and release of proinflammatory cytokines are the characteristics of pyroptosis [4,5,6]. Increasing evidence indicates that pyroptosis is involved in many diseases; however, the relevant mechanisms have not been fully understood [7]. Autophagy is an evolutionarily conserved lysosome-dependent catabolic process, in which cytoplasmic components, including protein aggregates, damaged organelles, and lipid droplets, are degraded, and their components are recovered. Autophagy plays an important role in maintaining intracellular homeostasis [8,9]. Autophagy has been reported to contribute to many diseases, such as cancer, cardiovascular diseases, metabolic diseases, neurodegenerative diseases, kidney disease, and lung disease [10]. Moreover, recent researches have shown that autophagy and pyroptosis together are involved in many pathophysiological processes [11,12]. However, the mechanism is not completely clear. Ischemia/reperfusion (I/R) injury refers to the phenomenon that the reperfusion of ischemic organs or tissues aggravates their injury [13]. I/R injury is characterized by many types of cell death, such as autophagy, apoptosis, necrosis, and necroptosis [14], indicating that inhibiting cell death can effectively improve I/R injury. Therefore, it is particularly important to clarify the mechanism of autophagy and pyroptosis in I/R injury, which can help to find ways to improve I/R injury. The role of autophagy and pyroptosis in I/R injury has not been reviewed. Therefore, in this review, we summarized recent progress about the role and the mechanism of autophagy and pyroptosis in I/R injury, hoping to provide a theoretical basis for further research in the future.

## 2. Overview of Pyroptosis

**Figure 1:**
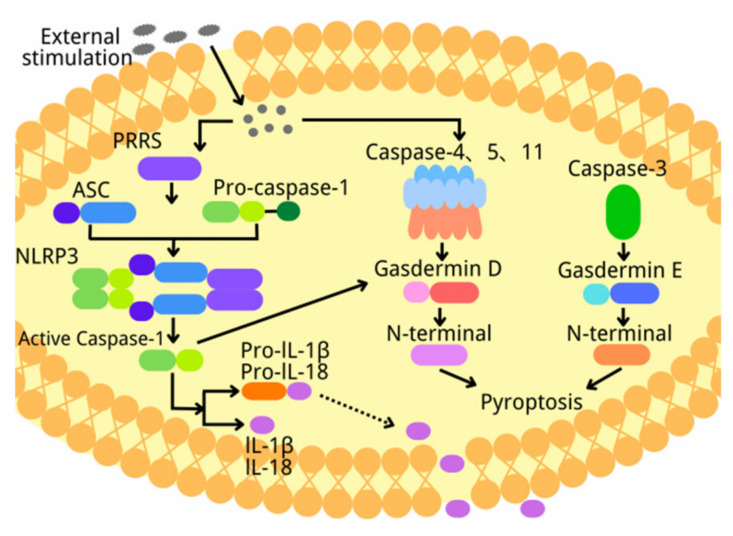
The process of three types of pyroptosis.

Brennan and Cookson found that Salmonella typhi can cause macrophage death through caspase-1, which is different from apoptosis. This kind of cell death was later named pyroptosis. Pyroptosis is different from apoptosis and necrosis in morphology and mechanism. During pyroptosis, the cell membrane is damaged to form perforations, the cells become swollen and ruptured, and inflammatory factors are released [15,16]. Pyroptosis is mediated by GSDM family proteins. The GSDM family includes six members: GSDM-A, -B, -C, -D, -E, and DFNB. Mice express only three GSDMAs (mGSDMA1–3) and four GSDMCs (mGSDMC1–4), but lack GSDMB. Except DFNB, all GSDM family members have a C-terminal self-inhibition domain, an N-terminal pore-forming domain, and a ring domain connecting the N-terminal and C-terminal domains. Protease-mediated cleavage in the linker ring releases the N-terminal domain, and then oligomerizes to form non-selective pores on the plasma membrane, resulting in membrane permeability changes, cell swelling, and membrane rupture [17,18]. Many pathological factors contribute to pyroptosis, such as inflammatory cytokines, oxidative stress, and cholesterol [19]. At present, according to different activation modes, there are three type of pyroptosis, namely, canonical pyroptosis pathway, non-canonical pyroptosis pathway, and caspase-3 mediated pyroptosis pathway [20,21]. Canonical pyroptosis is initiated by caspase-1 induced by NLRP3 inflammasome. The caspase-1 automatically cleaves into its p20/p10 dimer and CARD domain at its specific location. The two p20/p10 dimers then oligomerize to form a tetramer which cut the specific site of GSDMD. GSDMD is cleaved into N-terminal fragments which bind to the cell membrane to form the pyroptotic pore. P20/P10 tetramers then cleave pro-IL-1β and pro-IL-18 into IL-1β and IL-18. After that, water flows into cells due to the osmotic pressure to cause cell swelling. IL-1β and IL-18 then come out through GSDMD pores, leading to inflammation [7]. Similar to caspase-1, in non-canonical pyroptosis pathways, the activated caspase-4/5/11 cleaves GSDMD into N-GSDMD, which is transferred to the cell membrane and forms plasma membrane pores. Although caspase-4/5/11 cannot cleave pre-IL-1β/pre-IL-18, they can induce the maturation and secretion of IL-1β/IL-18 via the NLRP3/caspase-1 pathway. Moreover, GSDMD is cleaved by caspase-4/5/11, which also leads to K^+^ outflow to activate NLRP3 inflammasome, leading to inflammation [22]. In caspase-3-mediated pyroptosis pathway, caspase-3 cleaves GSDME and promotes the binding of GSDME-N domain to the cell membrane, thus inducing the formation of cell pores to lead to pyroptosis (Figure 1) [7,19,23,24]. More and more evidence shows that pyroptosis is involved in many diseases, including I/R injury [25,26,27]. However, the mechanism needs to be further clarified.

## 3. Overview of Autophagy

**Figure 2:**
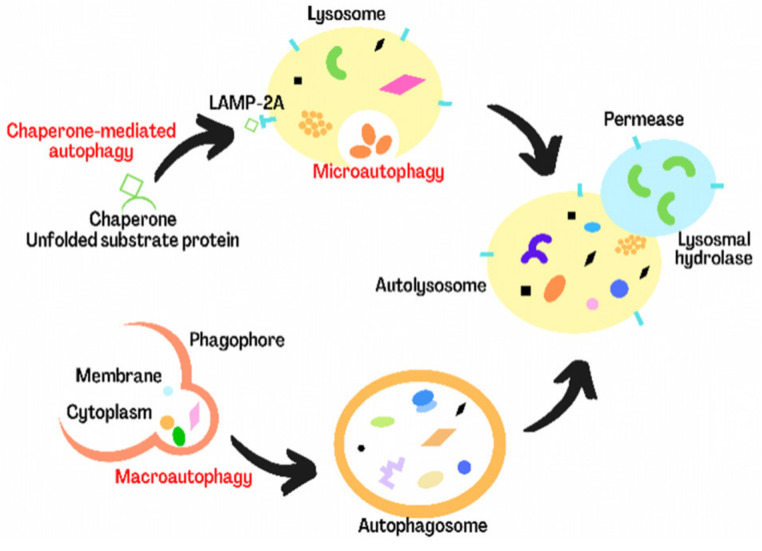
The process of three types of autophagy.

Autophagy is an important catabolic process through which cellular components, including proteins, lipids, and organelles, are degraded in lysosomes and then recycled, maintaining cellular homeostasis [9,28]. In the process of autophagy, the abnormal organelles and proteins, as well as pathogens, are first encapsulated in autophagosomes composed of double membranes and then transported to lysosomes for degradation [29]. At present, there are three kinds of autophagy in mammalian cells: macroautophagy, microautophagy, and chaperone-mediated autophagy [30]. Macroautophagy is the most studied autophagy, which mainly degrades microorganisms and organelles. In this process, the substance to be degraded is encapsulated in a double membrane vesicle to form autophagosome, which then fuses with lysosome to degrade its contents. Microautophagy mainly degrades cell components through embedding or dividing cytoplasm on lysosomal membrane. Chaperone-mediated autophagy is a kind of selective autophagy, in which intracellular proteins bound to chaperones are transferred to lysosomes for degradation (Figure 2) [7,31]. Under physiological conditions, autophagy is maintained at the basic level. Autophagy can also be significantly upregulated by many environmental stresses, such as nutrient deficiency, hypoxia DNA damage, cytotoxic agents, and growth factor deficiency, to alleviate the stress-induced damage and help cell survival [32]. If autophagy is maintained at a high level, it will induce cell death [33,34]. In recent years, more and more evidence demonstrates that dysfunctional autophagy is related with many diseases, including ischemia/reperfusion injury. However, the related mechanisms are not fully clear [35,36,37].

## 4. The Role of Pyroptosis and Autophagy in Ischemia/Reperfusion Injury of Nervous System

### 4.1. The Role of Pyroptosis and Autophagy in Cerebral Ischemia/Reperfusion Injury

Cerebral artery occlusion is the cause of the most ischemic strokes, leading to a significant increase in the global incidence rate and mortality of stroke. Diabetes is the main risk factor of ischemic stroke, which can increase its incidence rate by 1.8 to nearly 6 times and cause the poor prognosis after stroke [38,39]. Diabetes not only changes the structure of cerebral arteries, leading to their adverse remodeling and pathological neovascularization and vascular degeneration, but also changes the function of cerebral vessels, resulting in decreased myogenic reactivity and endothelial dysfunction. In addition, diabetes can also destroy the integrity of blood–brain barrier (BBB), resulting in rapid changes in blood flow and microbleeds entering the brain [40]. The mechanism of these changes by diabetes may be related to mitochondrial dysfunction, oxidative stress, and apoptosis [41,42]. Hypothermia has been reported to play a protective role in ischemic stroke [43,44]. However, the effect of hyperthermia on cerebral I/R injury aggravated by diabetes has not been clarified. To study this, Yanling Tu and colleagues established an in vivo model of diabetes cerebral ischemia by permanently blocking the middle cerebral artery (pMCAO) in rats with type 2 diabetes induced by a high-fat diet combined with intraperitoneal injection of STZ. Moreover, the cell model of diabetes cerebral ischemia was established by high glucose stimulation and oxygen glucose deprivation/reoxygenation (OGD/R) in vitro. The results of subsequent experiments showed that diabetes enlarged the volume of the cerebral infarction and brain edema, worsened the neurological deficit after cerebral ischemia, and increased the permeability of the blood–brain barrier (BBB). Mild hypothermia could improve these symptoms. Diabetes could increase BBB permeability by inducing the expression of MMP-9 and degrading the tight junction protein, while mild hypothermia abolished this phenomenon. Compared with the non-diabetes cerebral I/R injury model in vivo and in vitro, the expression of NOD-like receptor protein 3 (NLRP3), GSDM-N, cleaved caspase-1 and p62 in the diabetes cerebral I/R injury model increased, and the ratio of microtubule associated protein 1 light chain 3B (LC3B) II/I decreased; these effects were reversed by mild hypothermia, indicating that mild hypothermia upregulated autophagy and suppressed pyroptosis. Also, 3-MA (an autophagy inhibitor) decreased LC3II/I ratio and increased the expression of p62, NLRP3, GSDM-N, and cleaved caspase-1, indicating that mild hypothermia inhibited pyroptosis by promoting autophagy. Baf (an inhibitor of the combination of autophagosome and lysosome) could not notably upregulated the expression of NLRP3, caspase-1, and GSDM-N suppressed by mild hypothermia, suggesting that mild hypothermia can not inhibit pyroptosis through activating autophagy via inducing the fusion of autophagosome and lysosome. Collectively, mild hypothermia-improved diabetes aggravated cerebral I/R injury through suppressing pyroptosis by promoting autophagy [45]. Since 3-MA inhibits autophagosomes formation [46], therefore, in the above study, mild hypothermia upregulated autophagy via promoting autophagosomes formation. In addition, the activated autophagy aggravates the neuronal damage after pMCAO [47], which contradicts the conclusions of the above study. The reason may be related to the different time of MCAO, which needs to be further studied. Furthermore, NLRP3 inflammasome mediated autophagy regulation of pyroptosis in the above study. The mechanism of autophagy regulating NLRP3 inflammasome remains to be clarified.

Contrary to the above conclusion that the activating autophagy contributes to the neuroprotection against cerebral I/R injury, autophagy inhibition can also alleviate cerebral I/R injury. USP13 stabilizes the beclin1 subunit of Vps34-beclin1 and promote autophagy. Hence, USP13 inhibition suppressed autophagy [48,49,50]. Spautin-1 can suppress USP13 and is reported as an inhibitor of the early-stage autophagy [51]. Hui Liu and colleagues established in vivo and in vitro cerebral I/R injury models in male Sprague Dawley rats with 60 min of middle cerebral artery occlusion and 24 h of reperfusion or PC12 cell with OGD/R. In vivo experimental results showed that spautin-1 mitigated cerebral I/R injury by decreasing the infarct size and water content and improving the neurological impairment induced by cerebral I/R. Cerebral I/R significantly reduced the expressions of NLRP3, USP13, beclin 1, and GSDMD-N, which was abolished by spautin-1, indicating that spautin-1 inhibited autophagy and NLRP3 inflammasome-mediated pyroptosis. In vitro experiments demonstrated that PC12 cells with OGD/R manifested decreased cell viability and increased ROS, which was dampened by spautin-1. Moreover, spautin-1 could inhibit autophagy and NLRP3 inflammasome-mediated pyroptosis, evidenced by the reduction of the expression of autophagy and pyroptosis-related proteins in an in vitro I/R model. However, USP13 overexpression abolished these beneficial effects of spautin-1 and notably counteracted the spautin-1 inhibition of autophagy and NLRP3 inflammasome-mediated pyroptosis. Summarily, spautin-1 improved cerebral I/R injury through inhibiting autophagy and NLRP3 inflammasome-mediated pyroptosis [52]. ROS has been reported to play an important role in autophagy, NLRP3 inflammasome activation, and pyroptosis [53,54]. Therefore, ROS/NLRP3 inflammasome may mediate the effect of autophagy on pyroptosis in the above study. In addition, it can be seen from the above studies that sometimes the upregulated autophagy is beneficial to cerebral I/R injury, and sometimes it is the opposite. The reason may be that the level of autophagy is different. As we all know, the moderate autophagy has a protective effect on cells, while excessive autophagy has the opposite effect.

### 4.2. The Role of Pyroptosis and Autophagy in Spinal Cord Ischemia Reperfusion Injury

Spinal cord I/R injury (SCIRI) is one of the common spinal cord injuries, which can lead to neurological dysfunction and paralysis and seriously affect the quality of patient life [55,56]. There are currently a variety of treatments to prevent/treat SCIRI, including improved surgical techniques, hypothermia, and drug assistance [57,58]; however, there is still a lack of effective measures to prevent/treat SCIRI. Therefore, it is urgent to study the mechanism of SCIRI and then find an effective treatment for it [59]. Baicalein is a component of the roots of the herb Scutellaria baicalensis, which has anti-tumor, antibacterial, antiviral, antioxidant, and anti-inflammatory pharmacological effects [60,61]. It has been reported that baicalein can reduce I/R injury [62,63]. However, whether it can reduce SCIRI is not clear. The results of Chenyu Wu et al. showed that SCIR decreased the level of LC3II, beclin1, GRP78, ATF4, CHOP, CASP12 NLRP3, GSDMD, C-CASP1, IL1β, and IL18, and increased p62 level at the spinal cord lesion with SCIR, which were abolished by baicalein, indicating that baicalein promoted autophagy, inhibited endoplasmic reticulum (ER) stress, and pyroptosis. Baicalein also alleviated ER stress-mediated apoptosis. Furthermore, the inhibition of autophagy abolished baicalein suppression of ER stress-mediated apoptosis and pyroptosis induced by SCIR. Summarily, baicalein inhibited pyroptosis and ER stress-mediated apoptosis to improve SCIR by promoting autophagy [64]. In the above study, NLRP3 inflammasome mediated autophagy inhibition of pyroptosis. Evidence indicates that ER stress regulates pyroptosis [65,66]. The inhibition of ATF6 can induce NLRP3 inflammasome activation to further promote pyroptosis [67]. In baicalein improvement of SCIRI, whether ER stress regulates pyroptosis remains to be clarified. Autophagy, pyroptosis, and apoptosis are three important forms of cell death, which play an important role in many diseases [68,69]. Therefore, the action mechanism of autophagy, pyroptosis, and apoptosis in SCI and their relationship are worth studying.

### 4.3. The Role of Pyroptosis and Autophagy in Microglial with Oxygen-Glucose Deprivation/Reoxygenation

Neonatal hypoxic–ischemic brain damage (HIBD) is the main cause of morbidity and mortality in children, including epilepsy, cerebral palsy, and cognitive impairment [70,71]. At present, there is still a lack of satisfactory treatment for HIBD, which is a great threat to children’s health and quality of life. It is necessary to find effective methods to treat HIBD [72,73]. Mesenchymal stem-cell-derived exosomes (MSC-exos) have been reported to improve neonatal HIBD [74]. However, the relevant mechanism is not completely clear. The results of Zhenzhen Hu et al. showed that the identification of the shape, diameter, and surface marker protein of exosomes proved that MSC-exos were successfully extracted from human MSC. In OGD/R-induced BV-2 cells, MSC-exos upregulated cell viability, decreased the expression of NLRP3, cleaved caspase-1 and GSDMD-N, and caused IL-1β and IL-18 release, indicating that MSC-exos inhibited pyroptosis of BV-2 cells induced by OGD/R. Compared with cell-free conditioned medium (CM) from OGD/R-exposed BV-2 cells treated with PBS, CM from OGD/R-exposed BV-2 cells treated with MSC-exos notably upregulated SH-SY5Y cells viability and reduced LDH release, indicating that MSC-exos inhibited SH-SY5Y cell damage caused by OGD/R-induced BV-2 cells. MSC-exos also upregulated the expression of mitophagy-related proteins, including TOM20 and COX IV, in OGD/R-induced BV-2 cells. Meanwhile, 3-MA and mdi-1(mitochondrial division inhibitor-1) mitigated MSC-exo inhibition of pyroptosis, indicating that MSC-exos inhibited OGD/R-induced pyroptosis of BV-2 Cells through promoting mitophagy. In addition, FOXO3a siRNA partially reversed MSC-exos neuroprotection and mitigated MSC-exo-induced inhibition of mitophagy and pyroptosis, indicating that MSC-exos upregulated mitophagy to inhibit OGD/R-induced pyroptosis of BV-2 cells through promoting FOXO3a. Collectively, MSC-exos attenuated I/R-induced pyroptosis of microglia through upregulating FOXO3a expression to enhance mitophagy [75]. Mitophagy maintains the stability of the intracellular environment by selectively scavenging damaged mitochondria [76]. Hence, it can inhibits NLRP3 inflammasome activation through reducing mitochondrial ROS, which is an activator of NLRP3 inflammasome [77]. Therefore, it can be deduced that mitophagy inhibits the pyroptosis of microglia by suppressing NLRP3 inflammasome in the above study. FOXO3a has been reported to promote mitophagy through regulating mitophagy associated proteins, such as Parkin and BNIP3 [78]. Whether FOXO3a regulates mitophagy through the Parkin/BNIP3 pathway in MSC-exos neuroprotection deserves investigation in future.

## 5. The Role of Pyroptosis and Autophagy in Myocardial Ischemia and Reperfusion Injury

Myocardial I/R injury is a disease that leads to myocardial dysfunction, structural damage, and electrical activity disorder after the recovery of coronary blood flow in ischemic heart disease. It leads to a notable increase in the death rate of myocardial infarction and has become one of the main risk factors threatening human health [79,80]. Si-Miao-Yong-An decoction (SMYAD) is a kind of traditional Chinese medicine that is mainly used to clear away heat, detoxify, promote blood circulation, and relieve pain [81]. SMYAD has been reported to treat ischemic cardiovascular disease. However, the related mechanisms are not fully understood [82]. Wenwen Cui and colleagues found that SMYAD could alleviate the cardiac function injury induced by I/R in rats by improving ventricular volume and ejection fraction. Morphological analysis showed that SMYAD inhibited the formation of scar tissue and reduced fibrosis by downregulating the expression of collagen I and MMP9. In vitro experiments showed that SMYAD protected H9C2 cardiomyoblast cells from hyperoxia/reoxygenation (H/R). The in-depth experiment revealed that SMYAD upregulated the expression rate of LC3B-II/LC3B-I, reduced the expression rate of p-mTOR/mTOR, and downregulated the expression of NLRP3, caspase 1, and IL-1β in H/R cardiomyocytes, indicating that SMYAD activated autophagy and inhibited pyroptosis [83]. Our previous studies have shown that autophagy can be activated through the AMPK/mTOR pathway [84]. The mTOR signaling pathway has been reported to play an important role in myocardial I/R injury [85,86]. Therefore, it can be deduced that SMYAD improves myocardial I/R injury by promoting autophagy via mTOR pathway and inhibiting pyroptosis, which needs to use the inhibitor of autophagy and pyroptosisfor further validation. In the above study, autophagy may suppress pyroptosis via the inhibition of NLRP3 inflammasome. In addition, whether there is a regulatory relationship between autophagy and pyroptosis in the above studies remains to be clarified. Another study by Wenjing Sun et al. used overexpression of beclin1 in cardiac microvascular endothelial cells (CMECs) to study the effect of autophagy on pyroptosis during myocardial I/R injury. The results showed that beclin1 overexpression improved myocardial I/R injury in mice by reducing I/R-induced microvascular permeability and the myocardial infarct size. The in-depth study revealed that myocardial I/R induced caspase-4 activity and GSDMD expression, resulting in the increase of CMECs or heart pyroptosis mediated by caspase-4 in vivo and in vitro. Also, beclin1 overexpression in heart tissue and CMECs inhibited the activity of caspase-4 and reduced GSDMD level induced by I/R, thus downregulating caspase-4-mediated pyroptosis. Meanwhile, beclin1 overexpression also decreased I/R-induced IL-1β levels and promoted I/R-inhibited autophagy by increasing LC3II expression and decreasing p62 expression in the heart and CMECs. Further, 3-MA inhibited autophagy and abolished the inhibition of caspase-4-mediated proptosis by beclin1 overexpression in CMECs with H/R, indicating that overexpression of beclin1 suppressed caspase-4-mediated proptosis by promoting autophagy. Summarily, beclin1 overexpression ameliorated myocardial I/R injury by suppressing caspase-4-mediated pyroptosis by promoting autophagy [87]. In the above study, autophagy inhibits pyroptosis by suppressing caspase-4. In addition to caspase-4-mediated pyroptosis, there are also caspase-1 and caspase-3-mediated pyroptosis [7]. Therefore, whether beclin1 overexpression can inhibit the latter two types of pyroptosis in CMECs with H/R is worth studying in the future.

## 6. The Role of Pyroptosis and Autophagy in Limb Ischemia/Reperfusion-Induced Muscular Injury

Limb I/R injury is a life-threatening disease after the acute lower limb ischemia due to arterial embolism, trauma, primary thrombosis, limb or flap reattachment, arterial transplantation, and abdominal compartment syndrome [88,89,90]. Patients with mild limb I/R injury may have skeletal muscle fibrosis, permanent injury, and necrosis, while severe cases may develop multiple organ dysfunction syndrome [91,92]. Sulforaphane (SFN) is an organic sulfur compound that belongs to isothiocyanates and mainly exists in cruciferous vegetables [93,94]. One study shows that SFN has the functions of anti-tumor, anti-oxidation, anti-angiogenesis, and anti-inflammatory agents [95]. It has been reported that NLRP3-mediated inflammation and oxidative stress mediated by Nrf2 are involved in limb I/R injury [96]. Moreover, Nrf2 is an important target of SFN in many physiological and pathological processes [97,98,99]. The above suggests that SFN may improve limb I/R injury through Nrf2, which is confirmed by the research of Huanhuan Sun and his colleagues. The results showed that SFN mitigated limb I/R injury-induced muscular injury by improving muscle fiber degeneration, muscle edema, sarcoplasmic lysis, and inflammatory cell infiltration in mice. The in-depth study revealed that SFN reduced the secretion of inflammatory cytokine induced by I/R to improve inflammation in muscle of mice with limb I/R injury. Additionally, limb I/R injury upregulated the expression levels of pyroptosis-related proteins (NRP3, ASC, and caspase-1), LC3, and beclin-1 and downregulated p62 expression in mice, which were abolished by SFN, indicating that SFN suppressed limb I/R injury-induced pyroptosis and autophagy. In addition, SFN mitigated oxidative stress by reducing MDA production and increasing SOD activity in muscle of mice with limb I/R injury. Further mechanism studies confirmed that the antioxidant protection of SFN was related to the activation of Nrf2 are pathway [100]. The evidence indicates that Nrf2 pathway could modulate pyroptosis via ROS [101,102] and that ROS can promote autophagy [103]. Therefore, in the above study, it can be deduced that SFN may inhibit autophagy and pyroptosis by activating Nrf2 pathway to mitigate muscle injury caused by limb I/R injury, which needs to be further confirmed. In the protection of SFN, the relationship among pyroptosis, autophagy, and oxidative stress will be clarified in the future.

## 7. Conclusions

In this review, we summarized the role of autophagy and pyroptosis in different types of I/R injury as follows: (1) mild hypothermia improved cerebral I/R injury aggravated by diabetes by inhibiting pyroptosis via promoting autophagy; (2) spautin-1 improved cerebral I/R injury by inhibiting autophagy and NLRP3 inflammasome-mediated pyroptosis; (3) baicalein suppressed pyroptosis and ER stress-mediated apoptosis to ameliorate SCIR by promoting autophagy; (4) MSC-exos attenuated I/R-induced pyroptosis of microglia by increasing FOXO3a expression to enhance mitophagy; (5) SMYAD improves myocardial I/R injury by promoting autophagy via mTOR pathway and inhibiting pyroptosis, but the inhibitor of autophagy and pyroptosis is needed for further validation; (6) beclin1 overexpression ameliorated myocardial I/R injury by suppressing caspase-4-mediated pyroptosis via promoting autophagy; (7) SFN may inhibit pyroptosis by activating Nrf2 pathway to mitigate muscle injury caused by limb I/R injury, which needs to be further confirmed (Table 1).

Autophagy is a “double-edged sword” to cells. Moderate autophagy can clear damaged organelles and promote cell survival, but excessive autophagy may lead to cell death [31]. It can be seen from this review that autophagy occurs in I/R injury models of various target organs. Sometimes autophagy improves I/R injury, and sometimes it is the opposite. It is unclear whether autophagy plays a protective or destructive role after I/R, which may be related to the degree and persistence of autophagy activation, as well as the types and sources of cell or animal diseases. Similarly, pyroptosis has also proved to be a double-sided sword in different cases. On the one hand, moderate pyroptosis is conducive to maintaining cell homeostasis, improving immune activity, effectively removing damage and pathogens, and protecting the host. On the other hand, the excessive inflammation induced by pyroptosis is harmful to the host, which may promote the development of diseases, especially the progress of tumors, thus releasing various inflammatory factors and forming an inflammatory immune microenvironment [104]. At present, pyroptosis plays a promoting role in I/R injury. Whether pyroptosis can inhibit I/R injury remains to be studied.

It can be seen from the above that autophagy negatively regulates pyroptosis, mainly through NLRP3 inflammasome in some cases, during I/R injury, suggesting that there is a mutually exclusive relationship between autophagy and pyroptosis. In other cases, the two can also promote each other.This may be related to different types of stimulation or different stimulation time, because different stimulation or different stimulation time may lead to different levels of autophagy, which need further study.

It is believed that with the further development of related research, autophagy and pyroptosis will become important targets to improve I/R injury.

## Figures and Tables

**Table 1:** The summary of the role of autophagy and pyroptosis in ischemia reperfusion (I/R) injury.

The Type of Ischemia Reperfusion (I/R) Injury	The Role of Autophagy and Pyroptosis	Experimental Model	Reference
cerebral I/R injury aggravated by diabetes	mild hypothermia improved cerebral I/R injury aggravated by diabetes by inhibiting pyroptosis via promoting autophagy	type 2 diabetic rats/PC-12 cells with cerebral I/R injury	[45]
cerebral I/R injury	spautin-1 improved cerebral I/R injury by inhibiting autophagy and NLRP3 inflammasome-mediated pyroptosis	rats/PC-12 cells with cerebral I/R injury	[52]
spinal cord I/R injury (SCIRI)	baicalein suppressed pyroptosis and ER stress-mediated apoptosis to ameliorate SCIR by promoting autophagy	mice with SCIRI	[64]
microglial with oxygen-glucose deprivation/reoxygenation	MSC-exos attenuated I/R-induced pyroptosis of microglia by increasing FOXO3a expression to enhance mitophagy	oxygen-glucose deprivation/reperfusion-induced microglial	[75]
myocardial I/R injury	SMYAD improves myocardial I/R injury by promoting autophagy via mTOR pathway and inhibiting pyroptosis	mice/H9C2 cells with myocardial I/R injury	[83]
myocardial I/R injury	beclin1 overexpression ameliorated myocardial I/R injury by suppressing caspase-4-mediated pyroptosis by promoting autophagy	mice with myocardial I/R injury	[87]
limb I/R injury	SFN may inhibit autophagy and pyroptosis by activating Nrf2 pathway to mitigate muscle injury caused by limb I/R injury	mice with limb I/R injury	[101]

## Data Availability

Not applicable.
